# Long‐term skill improvement among general dental practitioners after a short training programme in diagnosing calcified carotid artery atheromas on panoramic radiographs

**DOI:** 10.1111/eje.12402

**Published:** 2018-11-12

**Authors:** Nils Gustafsson, Jan Ahlqvist, Eva Levring Jäghagen

**Affiliations:** ^1^ Oral and Maxillofacial Radiology, Department of Odontology Umeå University Umeå Sweden

**Keywords:** calcified carotid artery atheroma, carotid artery calcification, education, panoramic radiography, radiology

## Abstract

**Purpose:**

To study general dental practitioners (GDPs) ability to detect calcified carotid artery atheromas (CCAAs) in panoramic radiographs (PRs) and if their diagnostic accuracy in long term is improved after a short training programme.

**Methods:**

Fourteen GDPs had their diagnostic accuracy regarding CCAA in PR assessed at baseline, 2 weeks and 1 year after training. Comparison was made with a reference standard based on consensus results from two experienced oral and maxillofacial radiologists. At each session, 100 radiographs were assessed individually by the GDPs. After the baseline assessment, the GDPs participated in a 2‐hour training programme comprising a lecture and diagnostic training by calibration. The GDPs results before and after training were compared, as well as between follow‐up sessions.

**Results:**

A significant improvement in diagnostic accuracy was observed with increased sensitivity (from 41.8% to 55.7%, *P* = 0.02) without a significant decrease in specificity (from 87.2% to 86.7%, *P* = 0.87). The Kappa values also increased (from 0.66 to 0.71, *P* = 0.04). At 1‐year follow‐up, the improvement compared to baseline remained significant. There were no significant changes between the 2‐week and 1‐year follow‐up assessment.

**Conclusion:**

A short training programme can significantly and sustainable improve GDPs diagnostic accuracy regarding CCAA.

## 1 INTRODUCTION

According to the World Health Organization, heart disease and stroke are the two most common causes of death in the world in both middle‐ and high‐income countries,^1^ and can be prevented if detected early.^2^

**Figure 1:**
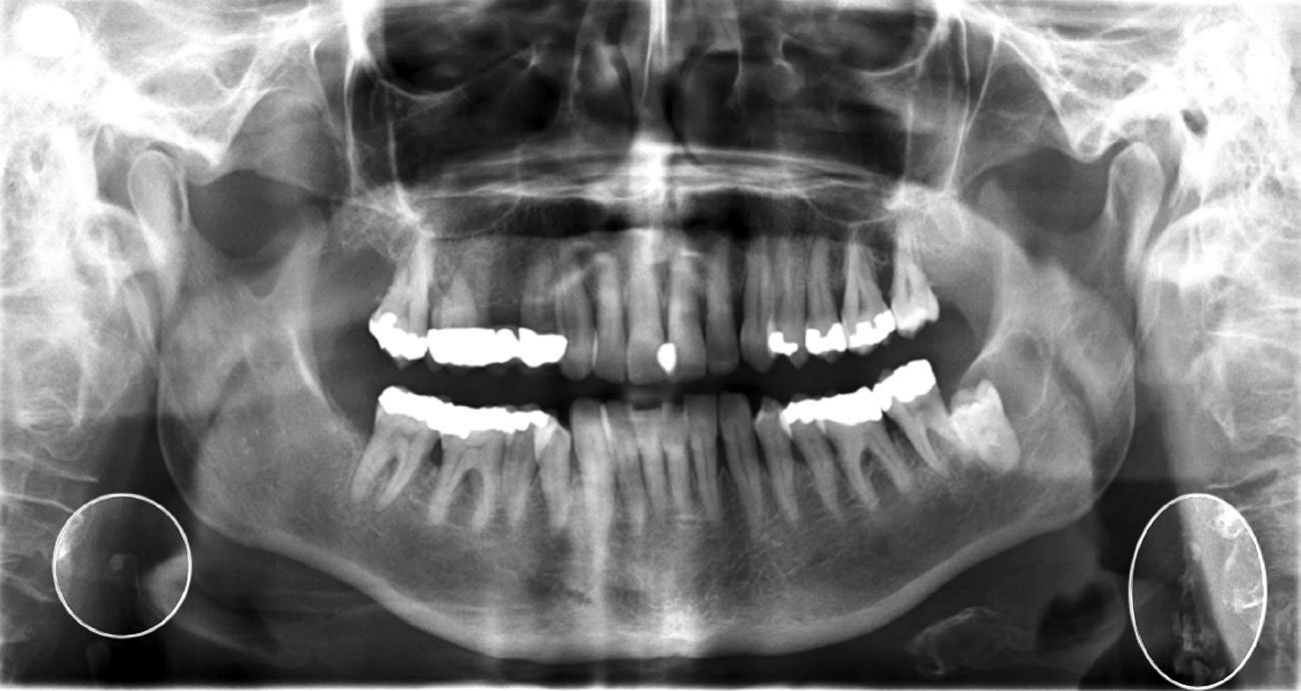
A panoramic radiograph depicting calcified carotid artery atheromas on the left and right sides

Many studies over the past decades have evaluated the diagnostic value of identifying calcified carotid artery atheromas (CCAAs) on panoramic radiographs (PRs) as a base for decisions regarding need for preventive treatment. (Figure 1). In recent years, the correlation between stroke,^3^, ^4^ myocardial infarction,^3^, ^5^, ^6^ diabetes^7^, ^8^ and CCAA have been strengthened suggesting that patients that present with CCAAs in PRs have an increased risk for cardiovascular events and should be referred to a physician for further examination and suitable preventive treatment.^9^, ^10^

Panoramic radiographs examinations are performed in both general and specialised dentistry. Incidental findings of CCAA on PR examinations, conducted for odontological reasons, could be a valuable tool for detecting those in need of further medical attention; that is, an assessment of risk for cardiovascular events and suitable prevention.^4^, ^9^, ^11^ A majority of all exposed PRs are assessed by general dental practitioners (GDPs). Implementation of diagnosing CCAA in general dentistry is desirable but requires that the GDPs have a high diagnostic accuracy, detecting high‐risk individuals with as few false positives as possible since that will burden the health care system without increasing survival.^9^ To facilitate that, GDPs need education and practice in diagnosing CCAA to gain high accuracy, particularly with high specificity.

Carotid artery atheromas diagnosed in PR by specialists in oral and maxillofacial radiology (OMFR) have a high association to carotid stenosis assessed with colour Doppler ultrasonography.^12^, ^13^ Their inter‐observer agreement is also high, with Kappa values between 0.69 and 0.88.^6^, ^13^, ^14^, ^15^, ^16^, ^17^, ^18^

To be able to correctly diagnose CCAAs, detailed knowledge about the bony anatomy, calcified cartilage and calcified pathological conditions in the area of the carotid artery is essential.^19^, ^20^, ^21^ However, diagnostic skill, defined as a high level of accuracy with high Kappavalues when compared to an expert, requires both theoretical knowledge and practical training with calibration.^22^, ^23^

We found only one study evaluating training for GDPs in diagnosing CCAAs in PRs and it included only one participant. The study showed that after completing a training package sponsored by the American Academy of Oral and Maxillofacial Radiology, the GDP had a high sensitivity but low specificity when diagnosing CCAA. The study did not include a long‐term follow‐up.^16^ Short training programmes including practical training have been shown to be effective to increased accuracy in diagnostic imaging among GDPs assessing osseous changes in the tempomandibular joint on Cone Beam Computer Tomography^24^ and among dental students assessing osteoporosis in dental radiographs.^25^ Similar results have also been reported for physicians when assessing pneumonia on chest radiographs^26^ and novices trained in doppler assessments of the intima thickness of the carotid artery.^27^ GDPs in general have a positive attitude to continuing education activities and find them beneficial and effective in improving their clinical practice.^28^

Our objective was as follows (i) to assess GDPs’ ability to detect CCAAs on PRs compared to consensus of two specialists in OMFR; (ii) to determine whether GDPs’ diagnostic abilities can be improved by means of a short, 2‐hour training programme, including a lecture and practical training with calibration of diagnosing and (iii) to investigate if improvements achieved in diagnostic accuracy are retained over time.

## 2 MATERIALS AND METHODS

### 2.1 General dental practitioners

Fourteen general dental practitioners (GDPs) provided informed consent. They had an average clinical experience of 7.1 years (ranging from 1 to 25 years). None of the participants had any specific training in diagnosing CCAA on PRs prior to inclusion, except what they had learned as undergraduates or through own independent studies of the literature. Twelve out of fourteen (86%) were aware of the possibility of diagnosing CCAA on PR, but only two considered themselves capable of diagnosing CCAAs on PRs. These two GDPs had assessed PRs for CCAA, but they had not been able to recognise CCAAs prior to the training session in this study. Seven of the GDPs had passed a course in panoramic imaging and seven were under training.

### 2.2 Sample of PRs

Panoramic radiographs were collected from a database of PRs performed for odontological reasons.^14^ All panoramic examinations had been performed at the department of Oral and Maxillofacial Radiology, Umeå University Hospital. All PRs were exposed in an Orthopantomograph^®^ OP100 (Instrumentarium, Tuusula, Finland), using the P1‐program. Fujifilm FCR, Fuji IP cassette type cc, size 15 × 30 (Fuji Photofilm Co., LTD, Tokyo, Japan) was used. An image reader (FCR Capsula XL; Fuji Photofilm Co., LTD, Tokyo, Japan) was used for scanning. Patients that presented CCAA had their degree of stenosis assessed with ultrasound of the carotid arteries but not for verification of calcifications since ultrasound is not the optimal method for that.^4^

The study material comprised fifty PRs with CCAA that were selected from the database using random inclusion^14^ (mean patient age, 66.6 years; min‐max, 45‐75 years). In addition, fifty PRs without CCAA were age and sex matched to those with CCAA. Another thirty PRs with CCAA and other calcified structures were selected from the remaining PRs in the database to be used for calibration. This image material had the same prevalence (50%) of CCAA, and also a high prevalence of other calcified anatomical structures. All PRs were anonymised and randomly included in a PowerPoint‐presentation without image compression to avoid reduced image quality.

All radiographs were assessed by two experienced specialists in OMFR (ELJ and JA), who previously had calibrated their assessments of CCAA in PRs to a high degree of agreement compared with computed tomography angiography and examinations of extirpated plaques. Their consensus was used as the expert assessment for this study.

### 2.3 Analysis of PRs

**Figure 2:**
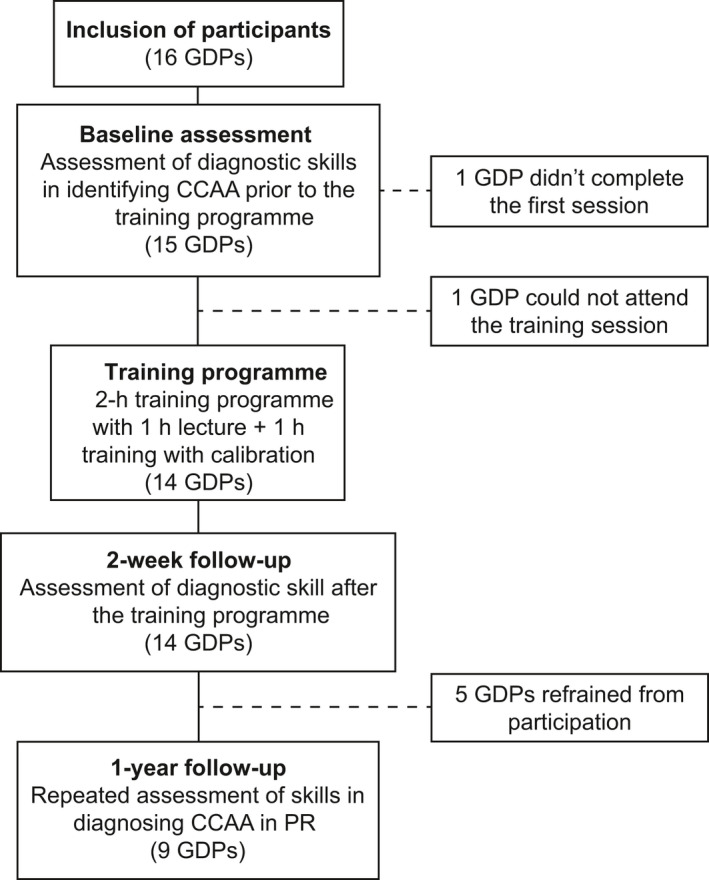
Flow chart of education programme and participant dropout

The analyses of the images were performed at three sessions: at baseline, and at a 2‐week and 1‐year follow‐up session. The latter to evaluate if there was a long‐term loss of acquired skills. The GDPs were blinded to all patients’ medical history, as well as to age and sex and the assessments of the PRs were performed individually. The task was to determine if a CCAA was present or not in the area of the carotid arteries on the PR. They all had a maximum of 2 hours to finalise the assessment per session. All participants finalised the assessments before the end of the session, except one who chose not to participate further. The first two assessment sessions were performed at the Department of Oral and Maxillofacial Radiology, Umeå University, in a dim‐lit room using full HD screens of high quality (HP ZR24w or HP Z24i) comparable to those commonly used in general practice. For the 1‐year follow‐up, one GDP chose to withdraw from participation. Four GDPs conducted their assessments at the department under the same conditions as the previous two sessions. The remaining nine GDPs were invited to conduct the assessment at their home clinics under dim lighting using diagnostic screens. Five of these nine (55%) returned their assessments. In total, nine participants completed the entire study protocol (Figure 2).

For all sessions, PowerPoint (Microsoft, Redmond, WA) was used to display the radiographs, which allowed zooming as well as adjustment of the contrast and brightness to improve interpretation of the CCAA in a manner similar to the software commonly used in general and specialised practice. During all sessions, the GDPs were allowed to change the contrast and brightness but they were not explicitly instructed to do so during the first session.

### 2.4 Training programme for GDPs

The GDPs participated in a 1‐hour lecture on panoramic imaging technique, anatomy and differential diagnostics of calcified structures in the area of the carotid artery in PRs. They were also trained in techniques for improving image interpretation of the digital PRs, such as zoom and changing the contrast and brightness levels.^19^, ^20^, ^29^ The lecture was given by two senior lecturers and OMFR specialists (ELJ and JA), both with more than 10 years of experience in diagnosing CCAA. After the lecture, the GDPs participated in a 1‐hour practical training session, which included assessing 30 PRs for calibration, and comparing their results with the consensus of expert assessments. The practical training was performed under the supervision of the authors. In total, the training programme lasted 2 hours. Different types of calcified structures in the area of the carotid arteries were presented in the 30 PRs, 50% of which included CCAA, to train the GDPs in differential diagnosis. The participants were allowed to discuss the training cases with fellow participants and specialists/teachers.

### 2.5 Statistics

The study was reported in accordance with Standards for Reporting of Diagnostic Accuracy Studies (STARD‐15)^30^ and Guidelines for Reporting Reliability and Agreement Studies (GRRAS).^31^ This includes calculations of sensitivity, specificity, positive and negative predictive value (PPV and NPV), accuracy (proportion correctly classified), positive and negative likelihood ratio (±LR). Inter‐observer agreement (Cohens Kappa) was calculated for each GDP compared to the expert assessment. Statistical Packages of Social Sciences (SPSS) 23 (IBM, New York, NY) was used for evaluation of means using analysis of variance (ANOVA), and post‐hoc analysis using Fishers Least Significant Difference (LSD) for comparisons between sessions. Change between baseline and the 2‐week follow‐up was also assessed with a paired t test and *P* < 0.05 was considered statistically significant.

### 2.6 Ethical considerations

The study was assessed by the Regional Ethics Committee of Umeå (Dnr: 2017/137‐31). They found no ethical issues that called for approval since all participants provided informed consent and all results analysed were anonymised.

## 3 RESULTS

Baseline assessments showed a mean sensitivity of 41.8% (min 8.8%, max 63.8%) and a mean specificity of 87.2% (min 65.0%, max 99.2%).Kappa values were estimated for each observer by comparison with the expert assessments (specialist consensus) resulting in a mean of 0.66 (min 0.51, max 0.75).

Comparisons between the mean values at baseline and at the 2‐week follow‐up showed a statistically significant increase in sensitivity from 41.8% to 55.7% (*P* = 0.05), without a significant decrease in specificity (87.2%‐86.7%; *P* = 0.88). The NPV had also increased significantly from 69.9% to 74.9% (*P* = 0.05). However, the increase in PPV from 67.6% to 75.8% was not statistically significant (*P* = 0.16). The mean Kappavalues increased from 0.66 to 0.71 (*P* = 0.04) after the training session. No change was seen in +LR; however, a significant decrease was seen for −LR from 0.67 to 0.51 (*P* = 0.01; Table 1).

**Table 1:** Mean values for each variable describing diagnostic accuracy with standard deviation (SD) and minimum (min) and maximum (max) values before and after training

Variables	Baseline			Two weeks follow‐up					ANOVA
	Mean (min‐max)	CI 95%	SD	Mean (min‐max)	CI 95%	SD	*P*‐values*	*P*‐values**	*P*‐values
Kappa	0.66 (0.51‐0.75)	0.62‐0.70	0.07	0.71 (0.60‐0.77)	0.67‐0.75	0.05	0.08	0.04	0.05
Sensitivity	41.8% (8.8%‐63.8%)	32.1%‐51.5%	18.2%	55.7% (33.8%‐71.3%)	46.0%‐65.4%	10.9%	0.05	0.02	0.05
Specificity	87.2% (65.0%‐99.2%)	82.5%‐91.9%	7.8%	86.7% (70.0%‐96.7%)	82.0%‐91.4%	8.8%	0.88	0.87	0.91
PPV	67.6% (25.9%‐94.7%)	59.3%‐75.8%	17.9%	75.8% (58.1%‐91.3%)	67.6%‐84.1%	10.2%	0.16	0.17	0.16
NPV	69.9% (57.8%‐78.2%)	66.2%‐73.5%	6.0%	74.9% (67.1%‐80.0%)	71.3%‐78.5%	3.5%	0.05	0.01	0.04
+LR	5.16 (0.53‐23.0)	1.97‐8.35	5.52	6.05 (2.08‐15.75)	3.85‐8.25	3.81	0.65	0.66	0.54
−LR	0.67 (0.41‐1.10)	0.54‐0.78	0.21	0.51 (0.38‐0.74)	0.45‐0.56	0.09	0.02	0.01	0.02

+LR, positive likelihood ratio; −LR, negative likelihood ratio; NPV, negative predictive value; PPV, positive predictive value.

* *P*‐values calculated using LSD post‐hoc comparing follow‐up session to baseline. *P* < 0.05 is considered significant.

** *P*‐values calculated using paired *t* test between the 2 wk follow‐up to baseline. *P* < 0.05 is considered significant.

At the 1‐year follow‐up, the increase in sensitivity and NPV compared to baseline remained statistically significant (*P* = 0.01 and 0.02, respectively). Kappa increased significantly from 0.66 to 0.74 between baseline and the 1‐year follow‐up (*P* = 0.01). Similarly, PPV increased significantly from 67.6% to 80.8% (*P* = 0.05) as well as NPV from 69.9% to 77.1% (*P* = 0.02). No change was seen in +LR; however, a significant decrease was seen for −LR from 0.67 to 0.46 (*P* = 0.01; Table 2).

**Table 2:** Mean values for each variable describing diagnostic accuracy with standard deviation (SD) and minimum (min) and maximum (max) values before and after training

Variables	Baseline			One year follow‐up					ANOVA
	Mean (min‐max)	CI 95%	SD	Mean (min‐max)	CI 95%	SD	*P*‐values*	*P*‐values**	*P*‐values
Kappa	0.66 (0.51‐0.75)	0.62‐0.70	0.07	0.74 (0.64‐0.99)	0.68‐0.79	0.10	0.01	0.26	0.05
Sensitivity	41.8% (8.8%‐63.8%)	32.1%‐51.5%	18.2%	57.8% (30.0%‐98.7%)	46.9%‐73.2%	19.2%	0.04	0.78	0.05
Specificity	87.2% (65.0%‐99.2%)	82.5%‐91.9%	7.8%	90.1% (69.2%‐100%)	82.0%‐94.8%	8.7%	0.44	0.36	0.91
PPV	67.6% (25.9%‐94.7%)	59.3%‐75.8%	17.9%	80.8% (61.5%‐100%)	68.6%‐90.9%	11.2%	0.05	0.44	0.16
NPV	69.9% (57.8%‐78.2%)	66.2%‐73.5%	6.0%	77.1% (67.5%‐99.2%)	72.5%‐82.3%	8.8%	0.02	0.44	0.04
+LR	5.16 (0.53‐23.0)	1.97‐8.35	5.52	7.72 (2.39‐22.50)	2.25‐13.20	6.54	0.27	0.47	0.54
−LR	0.67 (0.41‐1.10)	0.54‐0.78	0.21	0.46 (0.01‐0.76)	0.31‐0.62	0.20	0.01	0.61	0.02

+LR, positive likelihood ratio; −LR, negative likelihood ratio; NPV, negative predictive value; PPV, positive predictive value.

* *P*‐values calculated using LSD post‐hoc comparing follow‐up session to baseline. *P* < 0.05 is considered significant.

** *P*‐values calculated using LSD post‐hoc comparing the two follow‐up sessions. *P* < 0.05 is considered significant.

**Figure 3:**
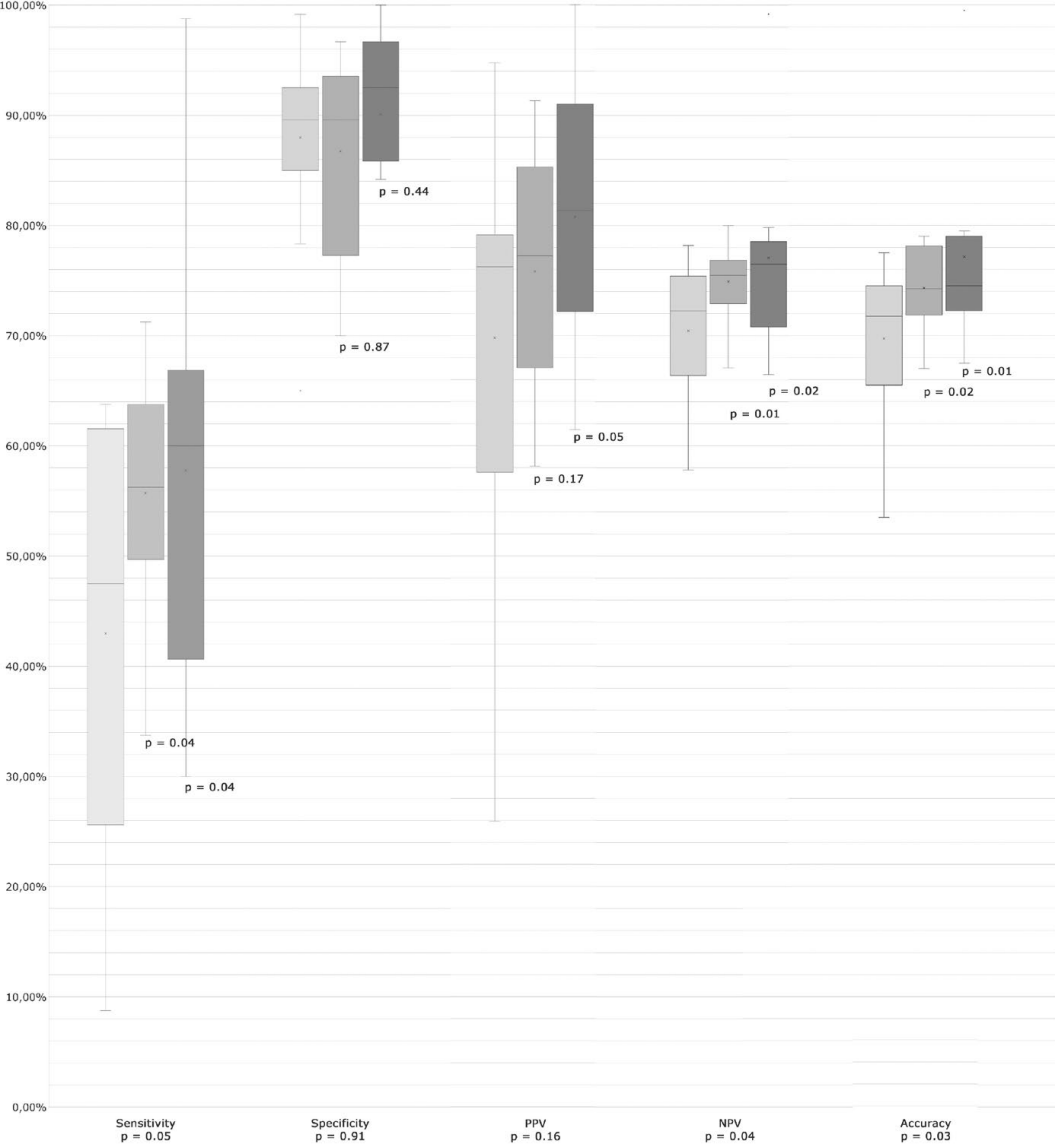
Comparison of participants’ diagnostic accuracy at baseline (light grey), the 2‐week follow‐up (medium grey) and the 1‐year follow‐up (dark grey) regarding sensitivity, specificity, positive predictive value (PPV), negative predictive value (NPV) and accuracy. The line in the box marks the median and × marks the mean.* P*‐values in the graph are for comparison of changes in means between follow‐up sessions and baseline, and *P*‐values on *x*‐axis are from the ANOVA comparing all three sessions

No statistically significant change was seen between the 2‐week and 1‐year follow‐ups for any of the studied variables (Table 2). Differences in diagnostic accuracy between the sessions are presented as a box‐plot in Figure 3, as well as in Tables 1 and 2.

During the year between baseline and the 1‐year follow‐up, all of the participants had reviewed PRs, however, specialists had made the final decision on diagnosis.

## 4 DISCUSSION

Our results indicate that GDPs have a low risk of false positive assessments even without specific training in CCAA diagnostics. After participating in a training programme including calibration, the sensitivity and Kappa values significantly increased, without a significant decrease in specificity. The GDPs also reached Kappa values corresponding to the lower spectrum (0.69‐0.88) reported in previous studies.^6^, ^13^, ^14^, ^16^, ^32^, ^33^ The increase in diagnostic accuracy was retained by the GDPs at the 1‐year follow‐up.

In a clinical situation, factors other than radiological findings can be considered by the GDPs before referring a patient to a physician, such as age, sex, medications, and previously known risk factors or cardiovascular disease. This makes it less likely that patients are unnecessarily referred and subjected to worry.^9^ To enable GDPs to give adequate referrals of people in need of medical attention, an increased sensitivity and specificity is desirable. However, considering the present situation where there is no common practice of diagnosing CCAA, many dental patients are not getting any attention for their CCAA. Any improvement in GDPs’ diagnostic accuracy in examining PRs could be beneficial to patients. We have shown that GDPs improve their sensitivity of diagnosis with continued high specificity, meaning that more CCAA can be detected, and patients with cardiovascular disease can be advised to seek further medical attention for preventive treatment, if GDPs are educated and utilise the information in the PRs by assessing CCAAs in their daily practice.

The prevalence of CCAA in the general population is most likely lower than in our study material. In the underlying database, the prevalence was 14% (ages 18‐75).^14^ However, this should not affect the generalisability of the training programme's effect, and since persons older than 60 years are more likely to benefit from an early diagnosis of CCAA and have a similar prevalence of CCAA as in our material,^34^ the results might be applicable to that group.

To minimise the false positive rate, the training programme focused on differential diagnostics and calibration on training cases, and also on the average location of CCAA. Different appearances of CCAA were also presented to increase awareness of different characteristics of calcifications in the area of the carotid arteries.^35^ Similar to our results, other studies of short training programmes on specific types of diagnostic imaging have reported improvements in diagnostic accuracy for GDPs,^24^ physicians^26^ and complete novices.^27^ The GDPs in the present study also presented high skill retention at the 1‐year follow‐up. Similar results have been found in studies of training of specific types of diagnostics among dental students, both using conventional and computer‐aided teaching models.^36^ The results suggest that a short training programme can be effective in improving accuracy in both short and long term.

Calcifications as small as 1 mm^3^ can be detected on PRs, and there are no correlations between the size of the calcification and the size of a possible stenosis, that is, carotid calcifications of any size can identify a patient in need of further medical attention.^35^ Evaluations of the GDPs results revealed that CCAAs that were not detected were usually either very small, located close to the outer boundary of the radiographs, or closely related to the cervical spine. This indicates that more attention should have been given to these parameters in the training programme and that future training programmes should further emphasise location and size in order to improve the outcome, and to increase the GDPs’ ability to detect these calcifications. Overall, a training programme with more practical training could increase the detection rate of calcifications that are more difficult to detect without deteriorating the specificity.

As seen in Figure 3, the GDPs had a wide range of diagnostic accuracy for CCAA compared to the expert assessments, both before and after participating in the training programme. Some participants reached a specificity level of over 90% combined with a sensitivity of over 90%. This may partially be explained by previous knowledge and/or experience in diagnosing CCAA on PR. High specificity was also found among five participants with low sensitivity; this was due to a very low level of registration of CCAA, with a lower risk of false positives and consequent lower chance of detecting CCAAs that were actually present.

The participating GDPs’ diagnostic accuracy of CCAA prior to the training programme was rather poor regarding detection of CCAA in PR. However, our study shows that their diagnostic accuracy was significantly increased after only 2 hours of training, which included a lecture and practical training with calibration. Previous studies of other diagnostic modalities and pathologies have shown that further training, including assessments of a larger number of patients, followed by feedback from experienced colleagues can improve the diagnostic accuracy further.^22^, ^26^ Our results indicate that patients with CCAA identified by a trained GDP would most likely have a calcification located in the carotid artery that might require medical attention.

Our results also suggest that GDPs after training have the necessary skills to diagnose CCAA on PRs, even though their knowledge at baseline was limited. Since participating in a short training programme significantly increased their skills, and since those skills were retained over time, it is likely that the low accuracy at baseline was due to lack of awareness of the structures in the area near the carotid artery that can appear on PRs rather than lack of skills.

### 4.1 Strengths and limitations

The strengths of this study include the opportunity to follow‐up on the GDPs’ diagnostic accuracy both after 2 weeks and in long term that is one year after participating in the training programme, as well as the possibility to offer a training programme provided by two senior specialists, with extensive experience in diagnosing CCAAs on PRs.

In this study, the consensus of two experienced specialists’ assessments was used as the expert assessments. Alternatives to the expert assessments could be the outcome of computed tomography angiography to determine whether calcifications are present in the carotid arteries.

A limitation is the small number of participants included in the study. A few withdrawals led to a high dropout rate which might have influenced the results.

The time for training was, approximately 2 hours, comprising a lecture, and practical training, with calibration. Further calibration sessions and discussions with specialists might have increased the inter‐observer agreement as reported in a previous study.^22^ A more qualitative evaluation of the training programme might also have been beneficial for further improvements of the educational quality.

## 5 CONCLUSION

A relatively short, 2‐hour training programme significantly and sustainable improved GDPs diagnostic accuracy regarding CCAA. The majority reached levels of sensitivity and specificity that provided a reasonable ratio of true positives to false positives. A longer training period, with supervision as well as repeated practical training sessions with calibration, may increase the sensitivity and specificity.

## CONFLICT OF INTEREST

None of the authors have any conflict of interests.
